# Laparoscopic Distal Gastrectomy Using Multimodal Visualization in a Patient with Situs Inversus Totalis and a Duplicated Portal Vein: A Case Report

**DOI:** 10.70352/scrj.cr.26-0196

**Published:** 2026-05-28

**Authors:** Takahiro Otani, Takuya Kichiraku, Satoru Motoyama, Kei Kotanagi, Rika Satoyoshi, Kazuhiro Kudo, Toshiya Sawada, Hideaki Miyazawa

**Affiliations:** Department of Gastrointestinal Surgery, Japanese Red Cross Akita Hospital, Akita, Akita, Japan

**Keywords:** duplicated portal vein, gastric cancer, laparoscopic gastrectomy, situs inversus totalis

## Abstract

**INTRODUCTION:**

Situs inversus totalis (SIT) is a rare congenital condition characterized by the complete mirror-image transposition of the thoracic and abdominal organs. Although arterial anomalies in SIT have been reported, venous anomalies, particularly those involving the portal venous system, are extremely rare and may increase the technical difficulty of minimally invasive surgery. We report a case of laparoscopic distal gastrectomy in a patient with SIT accompanied by a duplicated portal vein (DPV).

**CASE PRESENTATION:**

A 75-year-old woman with SIT presented with epigastric discomfort and was diagnosed with gastric cancer and multiple gallstones. Preoperative 3D-CT angiography revealed several vascular anomalies, including a DPV. Laparoscopic distal gastrectomy with D1+ lymphadenectomy and concomitant cholecystectomy was successfully performed using 3 surgical strategies. Preoperative 3D-CT evaluation enabled accurate identification of the vascular anatomy, and intraoperative multimodal visualization using 3 monitors facilitated safe orientation in the mirror-image operative field. Continuous communication between the surgeon and assistant further prevented anatomical misinterpretation. The operative time was 307 min, and blood loss was 50 mL. The postoperative course was uneventful, and the patient was discharged on POD 12. Histopathological examination revealed pT1b, pN0, cM0, and Stage IA gastric adenocarcinoma.

**CONCLUSIONS:**

Careful attention should be paid not only to mirror-image anatomy but also to vascular anomalies, particularly those of the venous system, in patients with SIT. Detailed preoperative and intraoperative assessments using multimodal visualization in conventional laparoscopic surgery may provide essential anatomical orientation, facilitating safe navigation, dissection, and precise intracorporeal reconstruction.

## Abbreviations

SIT
: situs inversus totalis
UICC
: Union for International Cancer Control

## INTRODUCTION

SIT is a rare congenital anomaly characterized by the complete right–left transposition of the thoracic and abdominal organs and is frequently associated with congenital cardiovascular and vascular abnormalities.^1)^ Although gastric cancer is uncommon in patients with SIT, several reports have demonstrated the feasibility of minimally invasive gastrectomy, with most studies focusing on arterial anatomical variations. Conversely, venous anomalies, particularly those involving the portal venous system, are rarely reported, and optimal surgical strategies for managing these complex anatomical variations remain unclear. Herein, we report a rare case of gastric cancer in a patient with SIT and a duplicated portal vein (DPV). Laparoscopic distal gastrectomy (LDG) was performed, including lymph node dissection and simultaneous laparoscopic cholecystectomy.

## CASE PRESENTATION

**Fig. 1:**
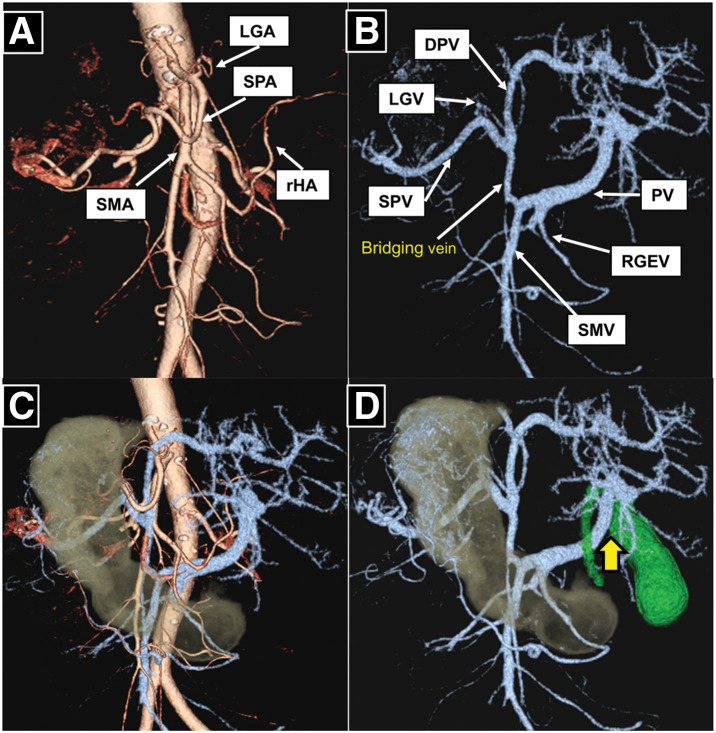
Preoperative 3D-CT images demonstrating reversed vascular anatomy. (**A**) Arterial anomalies included the absence of the CHA, with a rHA arising from the SMA. (**B**) A portal venous anomaly characterized by a DPV was observed. One PV originated from the SMV and the other from the SPV, with a bridging vein connecting the 2 vessels. (**C**) Schematic diagram showing the anatomical relationship between the stomach and the arteries and veins. (**D**) The PV was found to run ventral to the cystic duct (arrow). CHA, common hepatic artery; DPV, duplicated portal vein; LGA, left gastric artery; LGV, left gastric vein; PV, portal vein; RGEV, right gastroepiploic vein; rHA, replaced hepatic artery; SMA, superior mesenteric artery; SMV, superior mesenteric vein; SPA, splenic artery; SPV, splenic vein

A 75-year-old woman with SIT presented to our hospital with epigastric discomfort. Upper gastrointestinal endoscopy revealed a type 0–IIa lesion in the gastric antrum. CT revealed SIT with polysplenia and multiple gallstones. The patient was diagnosed with gastric cancer based on key indicators (L, Gre, 25 mm; tubular adenocarcinoma; cT1bN0M0; cStage I), as described in the UICC TNM classification (9th edition). Preoperative 3D-CT angiographic reconstruction revealed multiple vascular anomalies (**Fig. 1A**–**1D**). Arterial anomalies included the absence of the common hepatic artery (CHA) and a replaced hepatic artery arising from the superior mesenteric artery. In addition, a DPV was identified, with the splenic vein (SPV) and left gastric vein (LGV) draining into it. The right gastroepiploic vein drained directly into the portal vein (PV), which ran ventral to the cystic duct. Based on these findings, LDG with D1+ lymph node dissection and concomitant cholecystectomy was planned.

**Fig. 2:**
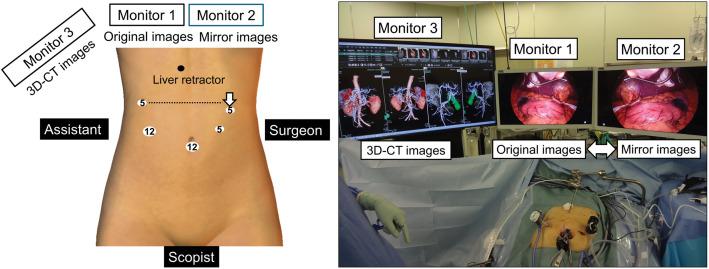
Port and monitor placement. Three monitors were placed at the cephalad side of the patient. The principal surgeon primarily stood on the left side and switched sides only during lymph node dissection of the pyloric antrum. Ports were arranged in a mirror-image configuration, with the 5-mm port in the left upper abdomen positioned slightly more caudally than usual. When the surgeon changed position, the monitor display was reversed accordingly.

To facilitate anatomical orientation, 3 monitors were placed on the cephalad side of the patient (**Fig. 2**): one displaying the original laparoscopic image, one providing a mirror-reversed image, and one showing the 3D-CT–based vascular reconstruction. The principal surgeon stood primarily on the left side and switched sides during lymph node dissection of the pyloric antrum. When the surgeons changed positions, the monitor display was reversed. A 2D rigid laparoscope (Karl Storz, Tuttlingen, Germany) was used, and the ports were arranged in a mirror-image configuration. The 5-mm port in the left upper abdomen was positioned slightly more caudally than usual, allowing the surgeon to perform suprapancreatic lymph node dissection using the dominant (right) hand.

**Fig. 3:**
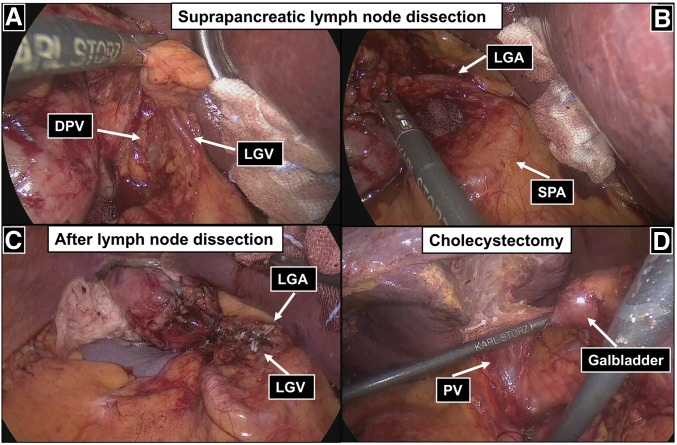
Intraoperative findings during suprapancreatic lymph node dissection and cholecystectomy. (**A**) The LGV drained into the DPV, coursing along the deep layer of the lesser curvature. (**B**) The CHA was absent. (**C**) Operative findings after completion of suprapancreatic lymph node dissection. (**D**) The PV ran ventral to the cystic duct. CHA, common hepatic artery; DPV, duplicated portal vein; LGA, left gastric artery; LGV, left gastric vein; PV, portal vein; SPA, splenic artery

During the suprapancreatic lymph node dissection, particular attention was paid to the vascular anomalies. The LGV, which drained into the DPV along the deep layer of the lesser curvature, was carefully divided while preserving the DPV (**Fig. 3A**). In the absence of the CHA, dissection of stations 8a and 9 was performed along the superior pancreatic border (**Fig. 3B**). The left gastric artery was subsequently divided to complete the dissection at station 7 (**Fig. 3C**). A cholecystectomy was performed after gastric resection. Because the PV ran ventral to the cystic duct, the gallbladder was transected at the neck using a linear stapler to avoid injury to either the bile duct or the cystic duct (**Fig. 3D**). A Billroth I reconstruction was performed (**Video S1**).

The total operative time was 307 min, with an estimated blood loss of 50 mL. The postoperative course was uneventful, and the patient was discharged on POD 12. Histopathological examination revealed pT1b (submucosal invasion), pN0 (0/25), and pStage IA disease based on the UICC TNM classification (9th edition).

## DISCUSSION

In previous cases of SIT, the focus has primarily been on arterial anatomical variations, whereas venous anomalies, particularly PV anomalies, have been rarely reported. However, venous anatomy may exhibit greater variability than arterial anatomy and is more susceptible to intraoperative injury and bleeding, necessitating meticulous intraoperative attention. To our knowledge, reports of minimally invasive gastrectomy in cases of gastric cancer with SIT accompanied by a DPV are extremely rare. DPV is a rare developmental anomaly characterized by the presence of 2 separate PVs ascending toward the porta hepatis.^2)^ In the present case, one PV originated from the superior mesenteric vein and the other from the SPV, with a bridging vein connecting the 2 vessels. In patients with SIT, given the mirror-image anatomy, such vascular anomalies may increase the risk of vascular injury during surgery if not recognized preoperatively, as a DPV may be misidentified as a minor collateral vessel or a thin vein and inadvertently injured, and the LGV draining into the DPV may be injured, particularly during suprapancreatic lymph node dissection. In this case, the PV ran ventral to the cystic duct. Significant inflammatory adhesions in Calot’s triangle, together with this anomalous venous course, may have precluded the safe achievement of the critical view of safety and rendered standard dissection of the cystic duct hazardous. To prioritize patient safety and avoid potential injury to the anomalous venous structures, we performed a subtotal cholecystectomy using a linear stapler at the neck, despite acknowledging the risks of retained stones and postoperative biliary complications. Therefore, careful preoperative assessment of vascular anatomy using high-resolution imaging is likely to be important, particularly in minimally invasive surgery.

In cases of SIT, surgeons should anticipate not only mirror-image organ positioning but also potential vascular anomalies. A systematic search of the English-language literature (PubMed) using the keywords “SIT,” “gastric cancer,” and “laparoscopic gastrectomy” or “robotic gastrectomy” from 2000 to 2025 identified 34 published cases (excluding the present case), including 22 laparoscopic and 12 robotic gastrectomies. Thus, a total of 35 cases (including the present case) were analyzed; vascular anomalies were found in 12 of these cases (34.3%) (**Table 1**).^3–13)^ Precise preoperative anatomical evaluation and careful intraoperative management, combined with reversed monitor displays and 3D imaging, enabled safe minimally invasive gastrectomy, despite the technical challenges posed by mirror-image anatomy and duplication of the PV.

**Table 1:** Previous reports of vascular anomalies in patients with SIT undergoing minimally invasive gastrectomy

Author	Year	Age, sex	Surgical approach	Gastrectomy	Lymph node dissection	Reconstruction	Surgeon position or port placement	Vascular anomaly	
								Artery	Vein
Fujikawa et al.^3)^	2013	60, F	Laparoscopic	Distal	D1+	Billroth I	Opposite	ALHA from the LGA	None
Min et al.^4)^	2013	52, M	Laparoscopic	Distal	D1+	Billroth I	Usual side	CHA from the SMA (rHA) RGA from the CA	None
								Two branches from the LGA	
Sumi et al.^5)^	2014	42, M	Laparoscopic	Distal	D1+	Billroth I	Opposite	LHA from the SMA	None
Shibata et al.^6)^	2018	79, M	Laparoscopic	Total	D2	Roux-en Y	Usual side	RGEA above the RGEV	None
Aisu et al.^7)^	2018	64, F	Robotic	Distal	D1+	Billroth I	Opposite	CHA and RGEA from the 1st jejunal artery	None
Namikawa et al.^8)^	2021	74, M	Laparoscopic	Distal	ND	Billroth I	Opposite	CHA from the SMA (rHA)	None
Fujita et al.^9)^	2022	67, M	Laparoscopic	Distal	D2	Roux-en Y	Opposite	CHA from the SMA (rHA)	None
Sagawa et al.^10)^	2022	64, M	Robotic	Distal	D1+	Billroth I	Usual side	LGA from the LHA	None
Zhu et al.^11)^	2023	63, M	Laparoscopic	Distal	D2	Billroth I	Usual side	RHA from the SMA (rRHA)	None
Cao et al.^12)^	2017	60, M	Robotic	Total	D2	Roux-en Y	NA	CHA from the SMA (rHA)	PV running anterior to the CA
								ALHA from the LGA	
Katano et al.^13)^	2022	62, M	Robotic	Proximal lower esophagectomy	D2, lower mediastinal LND	Esophago gastrostomy (SOFY)	Opposite	CHA from the SMA (rHA)	Two branches from the LGV
								ALHA from the LGA	
								Three branches from the LGA	
Our case		75, F	Laparoscopic	Distal cholecystectomy	D1+	Billroth I	Opposite	CHA from the SMA (rHA)	DPV present, SPV and LGV from the DPV, RGEV from the PV, PV running ventral to the cystic duct
								RGA from the rHA	

ALHA, accessory left hepatic artery; CA, celiac artery; CHA, common hepatic artery; DPV, duplicated portal vein; F, female; LGA, left gastric artery; LGV, left gastric vein; LHA, left hepatic artery; LND, lymph node dissection; M, male; NA, not available; PV, portal vein; RGA, right gastric artery; RGEA, right gastroepiploic artery; RGEV, right gastroepiploic vein; rHA, replaced hepatic artery; RHA, right hepatic artery; rRHA, replaced right hepatic artery; SIT, situs inversus totalis; SMA, superior mesenteric artery; SOFY, side overlap with fundoplication by Yamashita; SPV, splenic vein

To minimize vascular injury from anatomical misinterpretation, we implemented 3 key laparoscopic strategies. First, meticulous preoperative preparation using detailed 3D-CT evaluation of both arterial and venous anatomy identified patient-specific vascular anomalies. Additionally, reviewing mirror-reversed videos of standard gastrectomy procedures familiarized the team with mirror-image anatomy, and comprehensive information sharing ensured intraoperative safety.

Second, appropriate intraoperative strategies were required. In patients with SIT, port placement should be carefully planned to enable suprapancreatic lymph node dissection using the dominant hand of the surgeon. Multimodal visualization enabled real-time anatomical orientation and accurate identification of aberrant vascular structures. Dual-monitor systems with mirror-image displays have been previously reported in gastric and colorectal surgeries.^14–15)^ In the present case, an additional 3D-CT monitor was incorporated as an adjunct to further enhance intraoperative visualization.

Third, effective coordination between the surgeon and assistant was indispensable. Exchanging conventional grasping roles helped maintain familiar triangulation despite mirror-image anatomy. To avoid confusion, the surgeon primarily operated while viewing the original laparoscopic image and referred to the mirror-image display only when dividing critical anatomical structures. Conversely, the assistant primarily observed the mirror-image display and checked the original monitor only during active manipulation. Continuous communication and prompt feedback, together with real-time reference to the 3D-CT images, prevented anatomical misinterpretation. These strategies helped resolve visual disorientation for both the surgeon and assistant. This integrated approach reduced cognitive load and facilitated a more intuitive understanding of the operative field, thereby improving team coordination and enabling precise intracorporeal reconstruction, including safe stapling during anastomosis, with a level of accuracy that may be comparable to that achieved with robotic surgery.

Robotic surgery offers advantages in anatomically complex situations, including improved ergonomics, enhanced instrument articulation, and more intuitive hand–eye coordination.^16)^ In addition, it enables real-time assessment of vascular anatomy using 3D-CT angiography through image-guided surgery with the TilePro function^10)^ or image fusion, while the surgeon remains seated at the surgical console. This approach may be useful in complex cases. However, in unfamiliar operative fields such as those encountered in the present case, the close physical proximity between the surgeon and assistant in laparoscopic surgery may facilitate seamless communication and coordination compared to robotic platforms, where the console surgeon is physically separated from the bedside team. The present case suggests that multimodal visualization using conventional laparoscopy was a useful adjunct for improving spatial orientation and facilitating identification of critical structures. Simultaneous cross-referencing of original images, mirror-image views, and patient-specific vascular anatomy aided intraoperative understanding while preserving the simplicity and accessibility of conventional laparoscopic techniques. Although not equivalent to robotic systems, this multimodal visualization strategy provided essential anatomical orientation and facilitated safe dissection in complex SIT cases, even in a conventional laparoscopic setting, particularly where robotic-assisted surgery is unavailable or limited by cost, including at our institution. Analysis of additional cases in the future is required to validate the usefulness and generalizability of this multimodal visualization strategy in patients with SIT and complex vascular anomalies.

## CONCLUSIONS

In complex SIT cases, attention should be paid not only to mirror-image anatomy but also to vascular anomalies, particularly those involving the venous system. Careful pre- and intraoperative assessments using multimodal visualization in conventional laparoscopic surgery may provide essential anatomical orientation, thereby facilitating safe navigation, dissection, and precise intracorporeal reconstruction.

## SUPPLEMENTARY MATERIALS

Supplementary Video **Video S1** Laparoscopic distal gastrectomy with lymph node dissection and simultaneous laparoscopic cholecystectomy using multimodal visualization in a patient with SIT and a duplicated portal vein.
